# Human NGF “Painless” Ocular Delivery for Retinitis Pigmentosa: An In Vivo Study

**DOI:** 10.1523/ENEURO.0096-24.2024

**Published:** 2024-09-18

**Authors:** Debora Napoli, Noemi Orsini, Giulia Salamone, Maria Antonietta Calvello, Simona Capsoni, Antonino Cattaneo, Enrica Strettoi

**Affiliations:** ^1^CNR Neuroscience Institute, Pisa 56124, Italy; ^2^Regional Doctorate School in Neuroscience, University of Florence, Italy; ^3^Bio@SNS Laboratory of Biology, Scuola Normale Superiore, Pisa, Italy; ^4^Section of Human Physiology, Department of Neuroscience and Rehabilitation, University of Ferrara, Ferrara 44121, Italy; ^5^Rita Levi-Montalcini European Brain Research Institute (EBRI), Roma 00161, Italy

**Keywords:** blindness, cell survival, inherited retinal degeneration, microglia, neurotrophic factors, photoreceptors

## Abstract

Retinitis pigmentosa (RP) is a family of genetically heterogeneous diseases still without a cure. Despite the causative genetic mutation typically not expressed in cone photoreceptors, these cells inevitably degenerate following the primary death of rods, causing blindness. The reasons for the “bystander” degeneration of cones are presently unknown but decrement of survival factors, oxidative stress, and inflammation all play a role. Targeting these generalized biological processes represents a strategy to develop mutation-agnostic therapies for saving vision in large populations of RP individuals. A classical method to support neuronal survival is by employing neurotrophic factors, such as NGF. This study uses painless human NGF (hNGFp), a TrkA receptor-biased variant of the native molecule with lower affinity for nociceptors and limited activity as a pain inducer; the molecule has identical neurotrophic power of the native form but a reduced affinity for the p75NTR receptors, known to trigger apoptosis. hNGFp has a recognized activity on brain microglial cells, which are induced to a phenotype switch from a highly activated to a more homeostatic configuration. hNGFp was administered to RP-like mice in vivo with the aim of decreasing retinal inflammation and also providing retinal neuroprotection. However, the ability of this treatment to counteract the bystander degeneration of cones remained limited.

## Significance Statement

There is a huge need to develop treatments against cone degeneration in retinitis pigmentosa (RP) using mutation-independent approaches. We hypothesized that painless human NGF (hNGFp), a clinical-grade, validated NGF variant, might protect cones interacting with retinal microglia, as previously shown in the brain. However, we failed to achieve cone rescue by hNGFp employing various doses and routes on a mouse model of RP. It is possible that compared with brain microglia, retinal microglia are differently responsive to NGF or that cones are not sensitive to hNGFp-microglial effects. These negative results are relevant for the design of neurotrophin-based therapeutic strategies targeting retinal cells.

## Introduction

Retinitis pigmentosa (RP) is a family of genetic diseases causing retinal degeneration. The (several hundred) causative mutations are rod-specific in 98% of the cases and RP patients display nyctalopia as a first symptom. Progressively, cones, although not expressing the underlying mutation, also degenerate, leading to severe vision loss. Among the causes of the secondary (bystander) death of cones, recognized factors are the reduction in rod-derived cone viability factor (RdCVF; Clérin et al., 2023), necessary to cone survival, as well as the occurrence of retinal oxidative stress and chronic inflammatory responses (Campochiaro and Mir, 2018; Guadagni et al., 2019). These generalized biological processes are likely shared by different RP mutations; hence, their targeting represents a reasonable approach to disclose new therapeutic opportunities, counteracting the huge genetic variability of this disease, which limits the applicability of gene therapy. Reducing the bystander cone degeneration could save some diurnal vision, preserving life quality in numerous RP patients.[Table T1]

**Table 1. T1:** Mice used for the study

rd10 used in this study	Intravitreal IV hNGFp low dosage (0.54 5.4 ng/gr)	CTR (saline)	Intravitreal IV hNGFp high dosage (54 ng/gr)	CTR (saline)	Intravitreal IV NGF wt high dosage (54 ng/gr)	CTR (saline)	Intranasal hNGFp (0.54 ng/gr)	CTR (saline)
ICCH	8	7	8*	7*	6	6	4	4
qRT-PCR	9	8	8*	7*			13	13

*Numbers with an asterisk indicate mice whose eyes were used for multiple assays, i.e., for both ICCH and qRT-PCR.

Neuronal survival is classically sustained by neurotrophic factors (Huang and Reichardt, 2001; Skaper, 2018), with NGF as the prototypical neurotrophin (Levi-Montalcini, 1952, 1987). NGF activity is mediated by the tropomyosin receptor kinase/tyrosine kinase A (TrkA) and the p75NTR receptors, which elicit trophic or proapoptotic responses, respectively (Meakin and Shooter, 1992; Chao, 2003). Both receptors are expressed in different combinations/ratios in retinal cells, specifically in photoreceptors, Müller, and ganglion cells and in the retinal pigment epithelium (RPE; Carmignoto et al., 1991; Garcia et al., 2017; Rocco et al., 2021). Rodent retinal localization of TrkA/p75NTR is still debated: mRNA for NGF has not been detected in photoreceptors suggesting that they can receive NGF from other cells. NGF is secreted as a pro-NGF precursor and then cleaved by either intracellular or extracellular enzymes, yielding mature NGF homodimers. The signaling outcome on target neurons is influenced by the pro-NGF/NGF ratio, which is in favor of NGF during physiological conditions but switches toward pro-NGF during degenerative processes (Capsoni and Cattaneo, 2006; Iulita and Cuello, 2014; Ioannou and Fahnestock, 2017). Several studies and clinical trials have demonstrated the neuroprotective efficacy of NGF (Capsoni et al., 2011a; Aloe et al., 2015; Falsini et al., 2016a,b), and NGF eyedrops have been approved as a drug for corneal neurotrophic keratitis (Bonini et al., 2018). However, a therapeutic effect on RP patients has not been achieved. The dosage of the usable molecule is limited, as high levels of NGF target nociceptive receptors eliciting pain responses or p75NTR-related negative signaling.

A new pharmacological formulation was recently devised [painless human NGF (hNGFp)]. hNGFp carries an aminoacidic substitution that lowers its ability to induce pain, while maintaining its neurotrophic and neuroprotective action, in vitro and in vivo (Cattaneo and Capsoni, 2019). The binding affinity of hNGFp to TrkA remains high, while that for p75NTR is strongly reduced, limiting the capability of the molecule to trigger apoptosis (Covaceuszach et al., 2010; Capsoni et al., 2011b) . Interestingly, recent studies demonstrate an anti-inflammatory activity of hNGFp on human microglial cell lines which are induced to switch from a proinflammatory to a more homeostatic configuration (Cattaneo and Capsoni, 2019; Lisi et al., 2022). Hence, hNGFp, which combines a neurotrophic and a microglial homeostatic action, represents a hypothetically promising therapeutic tool to limit cone death in RP. To test the effects of this factor, we performed single intraocular injections of hNGFp in rd10 mutant mice (a known model of RP; Gargini et al., 2007), across the time window of maximum cone death, increasing progressively the dosage to attempt an amelioration of the retinal phenotype. The survival rate of cones and the retinal expression of inflammatory genes were assessed afterward. hNGFp was also repeatedly administered intranasally, as previously done to reach the retina or to target basal forebrain cholinergic neurons in Alzheimer’s disease mice (De Rosa et al., 2005; Capsoni et al., 2017). Yet, no rescue effects on the bystander degeneration of cones could be detected, suggesting a different sensitivity of retinal cells to hNGFp compared with other brain neurons and microglia.

## Materials and Methods

### Mice

The animals used for the study were homozygous rd10 mice (Pde6brd10/rd10) on a C57Bl6J background. They were originally purchased from the Jackson Laboratory and regularly bred in a local facility, where they were housed in standard cages in conformity with the current regulations on animal welfare. Water and food were provided *ad libitum*, ambient light was maintained below an average of 100 lux with a 12 h light/dark cycle, and room temperature was kept at 22°C. Animal experimental procedures were conducted in agreement with the current Italian and European laws and approved by the Italian Ministry of Health (Protocol no. 387/2020-PR, CNR Neuroscience Institute, Pisa, Italy); the experimental protocols were approved by the Ethical Committee of the CNR Neuroscience Institute. As a rule, 3–8 animals per experimental group were used, with male and female mice equally distributed. The administration of hNGFp/NGF or vehicle solution was performed by (1) intravitreal injection or (2) intranasal delivery. The intravitreal administration (Protocol 1) was done in animals at 50 d of age (P50) using a standard dosage of 0.54 ng/gr (hNGFp; in one set of experiments, a high dosage of 54 ng/gr hNGFp was used). The control solution was constituted by saline only; 1 µl of the solution was injected into each eye. For injection experiments, mice were deeply anesthetized with intraperitoneal injections of Zoletil 100 (80 mg/kg) and placed under a surgical microscope. Injections were performed manually with a 32-gauge needle connected by a Teflon tubing to a hydraulic microinjector; the solution was slowly delivered in the vitreous through the sclerocorneal margin. After 5 d, the animals were further anesthetized as above, and the retina was isolated. Retinal samples were used for real-time PCR or immunocytochemistry. An additional control experiment was performed as above replacing hNGFp with wild-type NGF at the dosage of 0.54 ng/gr.

In Protocol 2, the animals were treated 3 d/week beginning from age P25 until P45 or P60. In order to maintain a fixed dosage (0.54 ng/gr) and volume delivered, the concentration of the administered solution was weekly adjusted according to the animal weight. Seven drops of the experimental solution were administered alternately in each nostril (1 µl each) to awake mice. The control mice received saline solution only. Before starting the treatment, the animals were handled for a few days to accustom them to the experimental manipulation. A total number of 90 mice were used in the study, as specified in Table 1.

### Reagents

hproNGFp and hproNGFwt were produced as recombinant proteins in *Escherichia coli* and solubilized from inclusion bodies. After the chromatography steps, they were purified, and the precursors were cleaved by trypsin as previously described (Malerba et al., 2015). The mature forms were finally obtained.

### Retinal and retinal pigment epithelium (RPE) immunocytochemistry

The eyes were enucleated from mice from intranasal protocol, under deep Zoletil anesthesia; the animals were suppressed by cervical dislocation. The eyes were cut with an incision at the corneal margin and immersion-fixed for 30 min with 4% PFA in 0.1 M phosphate buffer (PB), pH 7.4, at RT; then, the anterior part, comprising the cornea and lens, was dissected out to obtain eyecups; the fixation step was completed for additional 30 min. The fixed eyecups were washed with PB (30 min) and cryoprotected with 30% sucrose in PB overnight, at 4°C; the eyecups were finally embedded in Tissue-Tek O.C.T. and frozen in dry-ice and cold isopentane for long-term storage at −20°C. Retinas were analyzed as whole mounts; additional retinal samples were sectioned vertically on a cryostat at 13−15 µm and collected on superfrost glass slides. RPEs were analyzed as flat mounts. For whole-mount immunocytochemistry (ICCH), the retina was gently dissociated from the sclera/choroid, also isolating the RPE; four radial cuts were done on the retinal surface while eight radial cuts were necessary to flatten the RPE. Two primary antibodies were used for retinal staining: anti-cone arrestin (rabbit polyclonal, diluted 1:1,000; catalog #ab15282, MilliporeSigma), to stain cones and anti-CD11b (rat monoclonal, diluted 1:500; catalog #ab8878, M1/70 Abcam), to label microglial cells. A blocking solution was done with PB plus 5% serum and 0.5% Triton X-100 (O/N 4°C) while primary and secondary Ab solutions were done with PB plus 1% serum and 0.1% Triton. A donkey anti-rabbit IgG-conjugated with Alexa Fluor 568 (diluted 1:500; catalog #A-11011, Invitrogen Thermo Fisher Scientific) and donkey anti-rat IgG-conjugated with Alexa Fluor-488 (diluted 1:500; catalog #712-546-153, Jackson ImmunoResearch) were used as secondary antibodies to reveal cones and microglia. Incubation with primary and secondary antibody solution was carried on for respectively 3 and 2 d. RPE flat mounts were processed in parallel with retinas using the same protocol, and the primary antibody anti-zonula occludens 1 (ZO-1) (rat polyclonal, diluted 1:500; catalog #MAB11, Merck) was revealed with an anti-rat Alexa Fluor-488  secondary antibody. For nuclear staining, retinas and RPEs were counterstained with Hoechst (0.02 mg/ml) for 30′ and finally mounted with Vectashield antifade mounting medium (catalog #H-1000-10, Vector Laboratories) on glass slides. For the retinal sections, the ICCH protocol was based on the same antibodies but had a shorter duration, with the primary antibody incubation carried on overnight at 4°C.

### Microscopy and image analysis

Images of the retina and RPE whole mounts were acquired with a Zeiss Imager.Z2 microscope equipped with an Apotome2 device (Carl Zeiss) and using a Plan Neofluar 40×, a numerical aperture (NA) of 1.25 oil objective; additional acquisitions were done with a Zeiss LSM 900 Confocal Microscope (Carl Zeiss), using a 40×/1.4 Plan Apochromat oil objective. For cell counting purposes, retinal samples with cone arrestin and CD11b staining were examined in whole mounts and sampled along four radial axes, choosing two midperipheral and two central locations to follow the photoreceptor degeneration pattern from the far periphery to the proximity of the optic nerve head. A total of 16 sampling fields were imaged for each retinal preparation; each field consisted of a *z*-stack encompassing the whole width of the outer nuclear layer (ONL). Reconstructions of the whole-mount samples were obtained by tile image acquisitions using a 10× objective. A similar imaging protocol was used for acquisitions of RPE flat mounts labeled by ZO-1 antibodies. RPEs were imaged along the four radial axes, from the most distal regions to the closest to the optic nerve head, obtaining a total of 20–24 images for each RPE. Optimally flattened areas were chosen, and maximum projections, bidimensional images, were obtained from single *z*-stacks. The Zeiss software ZEN 3.1 was used to adjust the brightness and contrast of the images, saved as tiff files.

For cone counts, each imaged retinal field was manually scored with MetaMorph software navigating through each plane of the *z*-stack until all cones in the stack were accounted for. Absolute and mean cell density were calculated and statistically compared using GraphPad Prism 8.0. The continuity of ZO-1 profiles was quantified by overlapping each RPE image with a custom grid (20 µm spaced) and manually counting with ImageJ the intersection points between the grid and the ZO-1–positive profiles. The mean and density of the obtained scores were statistically compared with GraphPad Prism 8.0. Whole-mount images of retinas were acquired with a Zeiss Imager.Z2 microscope equipped with an Apotome2 device (Carl Zeiss) and using a 10× objective. Lack of cone arrestin staining was considered a landmark of the degenerated area; the extension of this area was measured by ImageJ tools, normalized to the entire retinal extension, and expressed as percentage or mm^2^. Final data were analyzed with GraphPad Prism 8.0. Retinal sections stained with cone arrestin antibodies and counterstained with the nuclear dye DAPI were imaged using the Zeiss Imager.Z2/Apotome microscope utilizing a Plan Neofluar 20× objective with a numerical aperture (NA) of 0.8. Three retinal sections, located at the equatorial plane and containing the optic nerve head, were selected for each animal. The retinal sections were imaged by selecting six different points to include the central area, as well as midperipheral and peripheral eccentricities, located on opposite sides relative to the optic nerve head. The outer nuclear layer (ONL) of each image was measured using an ImageJ tool along a perpendicular line encompassing all the photoreceptor nuclei. Each measurement was repeated three times. The measurements were recorded in an Excel file and statistically compared using GraphPad Prism 8.0.

### RNA extraction and quantitative PCR

Total RNA was purified from whole rd10 retinal homogenates using an RNeasy Mini Kit (Qiagen) and following the manufacturer’s guidelines. A NanoDrop 2000 C spectrophotometer (Thermo Fisher Scientific) was used to determine the RNA concentration of each retinal sample. Two different kits of CDNA synthesis were used depending on the type of real-time protocol of choice. RT2 First Strand Kit was employed (catalog #330401, Qiagen) when gene expression was later quantified using ad hoc RT2 Profiler PCR Arrays. The QuantiTect Reverse Transcription Kit (catalog #205311) was used in combination with single gene expression TaqMan assays. All the real-time PCR reactions were performed and monitored using a Step One Plus machine (Thermo Fisher Scientific). Quantitative values for cDNA amplification were automatically calculated by the Step One Plus software from the threshold cycle number (Ct) obtained during the exponential growth of the PCR products. Data were analyzed by the ΔΔCt methods using housekeeping gene expression to normalize the cDNA levels of the transcripts under investigation. For real-time PCR arrays, data analysis was performed using the RT2 Profiler PCR Array data analysis template from Qiagen. Results were shown in a volcano graph where the fold change of each gene was plotted against its -log10(*p* values).

### List of genes analysis by RT2 Profiler PCR Arrays

P25–P45: spp1, tnfsf11, mfge8, gfap, cd68, tmem119, csf1, scl2a1, arg1, ccr2, ccl2, tnf, il1b, infg, myd88, lamp2, vegfa, mtor, il10, il2, itgam, aif1, mmp2, irf8, cybb, dhcr7, arsa, gaa, cxcl5,ccl12, nfkb1, nos2, cngb1, gnat1, gnat2, arr3, bcl2, bcl21, bax, bak1, vcam1, icam1, il6, actb, gapdh.

P25–P60: spp1, tnfsf11, mfge8, gfap, cd68, tmem119, csf1, csf1r, slc22a1, arg1, ccr2, tnf, ifngr1, ifngr2, myd88, lamp2, vegfa, mtor, il10, il2, itgam, il1b, nfkb1,nos2, cngb1, gnat1, gnat2, arr3, bcl2, bax, bak1, vcam1, icam1, cx3cr1, aif1, irf8, mmp2, arsa, ppard, cxcl5, ccl12, ccl2, c3cr1a.

TaqMan assays used for single gene expression: arr3 (Mm00504628_m1), gnat2 (Mm00492394_m1), gfap (Mm01253033_m1), tmem119 (Mm00525305_m1), arg1 (Mm00475988_m1), nos2 (Mm00440502_m1), myd88 (Mm00440338_m1), csf1 (Mm00432686_m1), nr3c1 (Mm00433832_m1), ccl2 (Mm00441242_m1), cxcl5 (Mm00436451_g1), ccl12 (Mm01617100_m1), lamp2 (Mm00495267_m1), ngfr (Mm00446296_m1), ntrk1 (Mm01219406_m1), actb (Mm02619580_g1).

#### Statistics

Average measurements from rd10 treated with hNGFp/NGF were compared with data obtained from matched control mice treated with only vehicle. The analyses were performed using an unpaired *t* test, and the significance value was set at ≤0.05. Data were analyzed with GraphPad Prism 8.0.2, which was also used to produce the graphical bar representations.

## Results

### Scope of the study

The scope of this study is to test the hypothesis that hNGFp can exert a rescue effect on the bystander degeneration of cones in a mouse model of RP by virtue of its demonstrated ability to direct microglial cells toward a homeostatic configuration and to exert a neuroprotective action on CNS neurons even when (as is the case for retinal cones) they do not express the NGF receptor TrkA. Indeed, single-cell RNA sequencing did not detect NTRK1 (the gene coding for TrkA) in mouse retinal cones, both in wt and in mice with photoreceptor degeneration (Karademir et al., 2022). Comparison of global gene expression between wt and Nrl^−/−^ mice (with an all-cone retina) did not show any increase in NTKR1 expression associated with the enormous number of extra cones of this knock-out (Brooks et al., 2011). Finally, immunohistochemical localization studies in the rodent retina did not show the presence of TrkA receptors in photoreceptors, although the antigen is present in other retinal cells (Telegina et al., 2019). Hence, in this study, both cone degeneration and putative cone rescue are supposed to take place by means of bystander interactions, likely mediated by microglial and/or other retinal neurons.

### The rd10 mutant mouse

**Figure 1.**
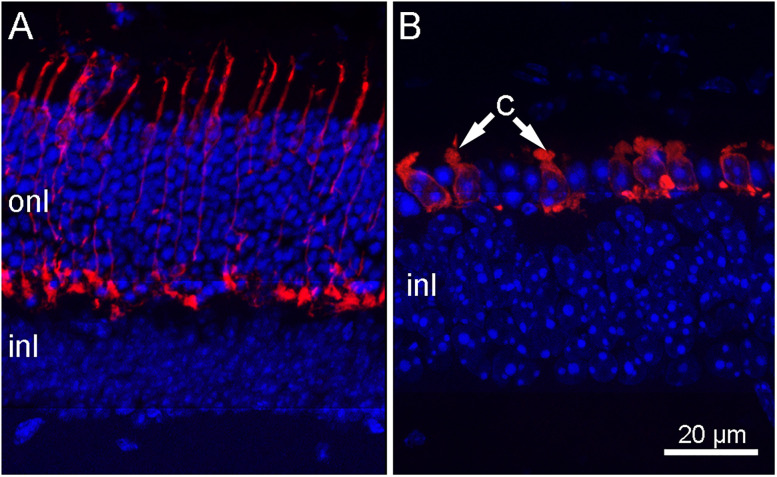
rd10 mutant mice retinal phenotype. Vertical sections of the outer retina of a typical C57Bl6 mouse (***A***), compared with that from a rd10 mutant (***B***) near the peak of photoreceptor degeneration (50 d of age). The nicely elongated, regularly spaced cones visible in ***A*** after cone arrestin antibody staining (in red) have largely degenerated and remodeled into hypertrophic cells with short outer segments (arrows) in B. Nuclei are stained blue with DAPI. onl and inl, outer and inner nuclear layers, respectively.

This well-known mutant, isolated at the Jackson Laboratory (Chang et al., 2007) and further characterized phenotypically (Gargini et al., 2007), carries a missense mutation of the rod-specific phosphodiesterase gene (PDE), conferring to the protein a partial loss of function. Compared to the other known PDE mutant (the rd1 mouse), the rd10 has a relatively slower loss of photoreceptors, starting around the third postnatal week, with a peak of rod death ∼24 d of life (P24). Cone loss is delayed, peaking near P45 (Fig. 1), thereby modeling the rod-cone degeneration of typical human RP. In this study, rd10 mice were treated with hNGFp at P50, across the window of maximum cone death, and deliberately attempting to achieve a pure rescue of cones, at the peak of cone loss and at a stage of largely completed rod degeneration.

### Single intravitreal injections of hNGFp show no consistent effects on late cone degeneration and expression of retinal proinflammatory markers in RP mice

**Figure 2.**
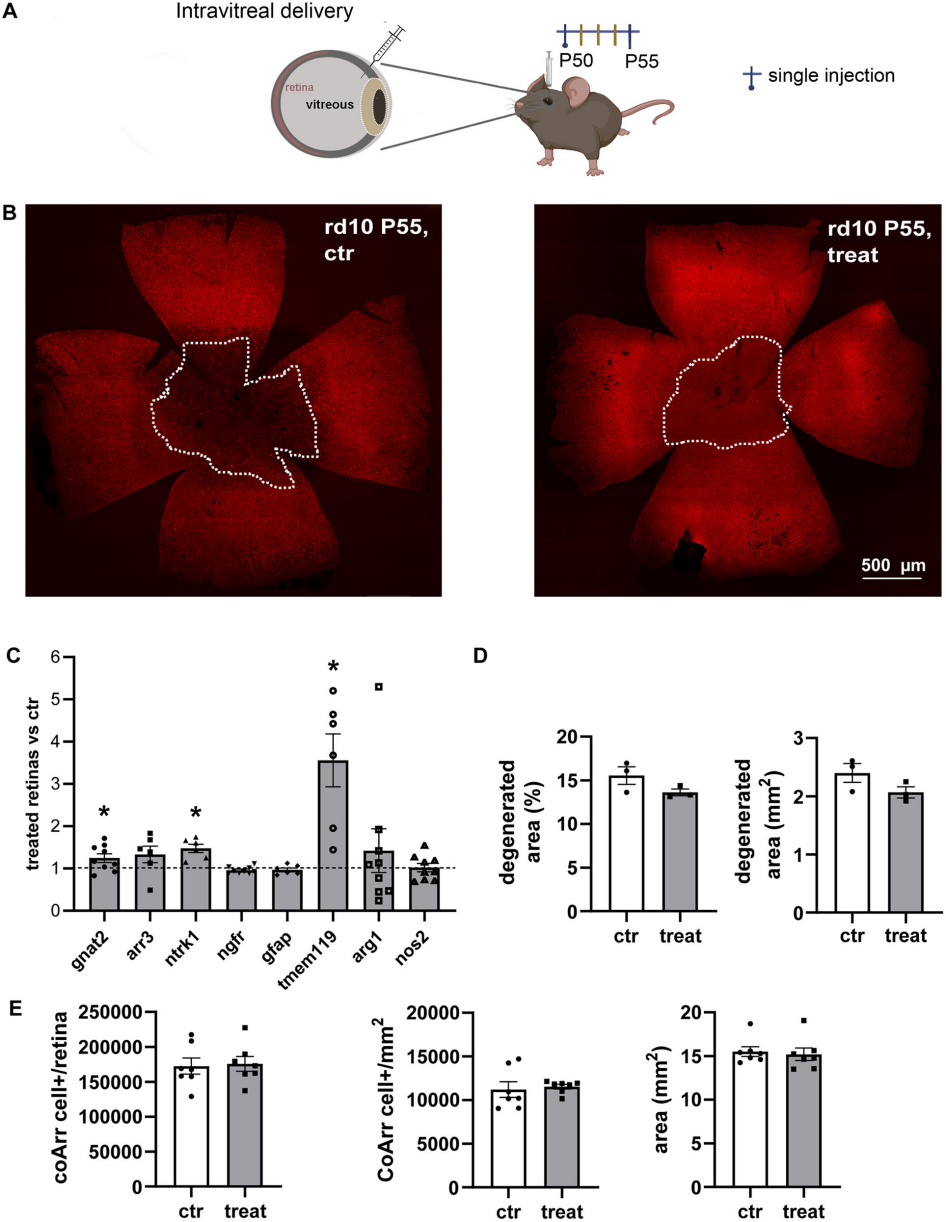
Intravitreal delivery of hNGFp has a moderate effect on cone survival in RP mice. ***A***, Experimental design used for the single intravitreal injection. The animals were divided into two groups as for the previous experiments, control (ctr) and hNGFp-treated (treat) and once intravitreal injected with PBS (ctr) or hNGFp (treat) at age P50; hNGFp dosage, 0.54 ng/gr. ***B***, Whole-mount retinas of control (left) and treated mice (right) stained with cone arrestin (red signal). The dotted lines define the degenerated area. ***C***, qPCR quantification of genes involved in photoreceptors survival and microglial activation. Gnat2 (ctr = 8, treat = 9; *p* = 0.050) and Arr3 (ctr = 5, treat = 6; *p* = 0.184) are genes expressed in rods and cones, ntrk1 (ctr = 5, treat = 6; val = 0.003) is the gene for trkA expression while ngfr (ctr = 8, treat = 9; *p* = 0.327) is the one for p75NTR receptor expression. Gfap (ctr = 5, treat = 6; *p* = 0.749) refers to macroglial activation while tmem119 (ctr = 5, treat = 6; *p* = 0.007) represent the activation of microglia. Arg1 (ctr = 8, treat = 9; *p* = 0.552) and nos2 (ctr = 8, treat = 9; *p* = 0.916) are, respectively, genes expressed by anti-/inflammatory and pro-/inflammatory microglia. Gene expression is reported as the ratio between the ctr and treat animals. Normalization was done on actin expression for each sample. ***D***, Degenerated area quantification expressed as mm2 (ctr = 3, treat = 3; *p* = 0.153) and percentage (ctr = 3, treat = 3; *p* = 0.146) with respect to the total retinal area. ***E***, Quantification of cone arrestin signal. Absolute cone number per retina (ctr = 7, treat = 8; *p* = 0.835); cone density (ctr = 7, treat = 8; *p* = 0.723); and retinal area (ctr = 7, treat = 8; *p* = 0.729). Error bars are ±SEM. *t* test was used to compare the ctr versus treat mean for each experiment.

**Figure 3.**
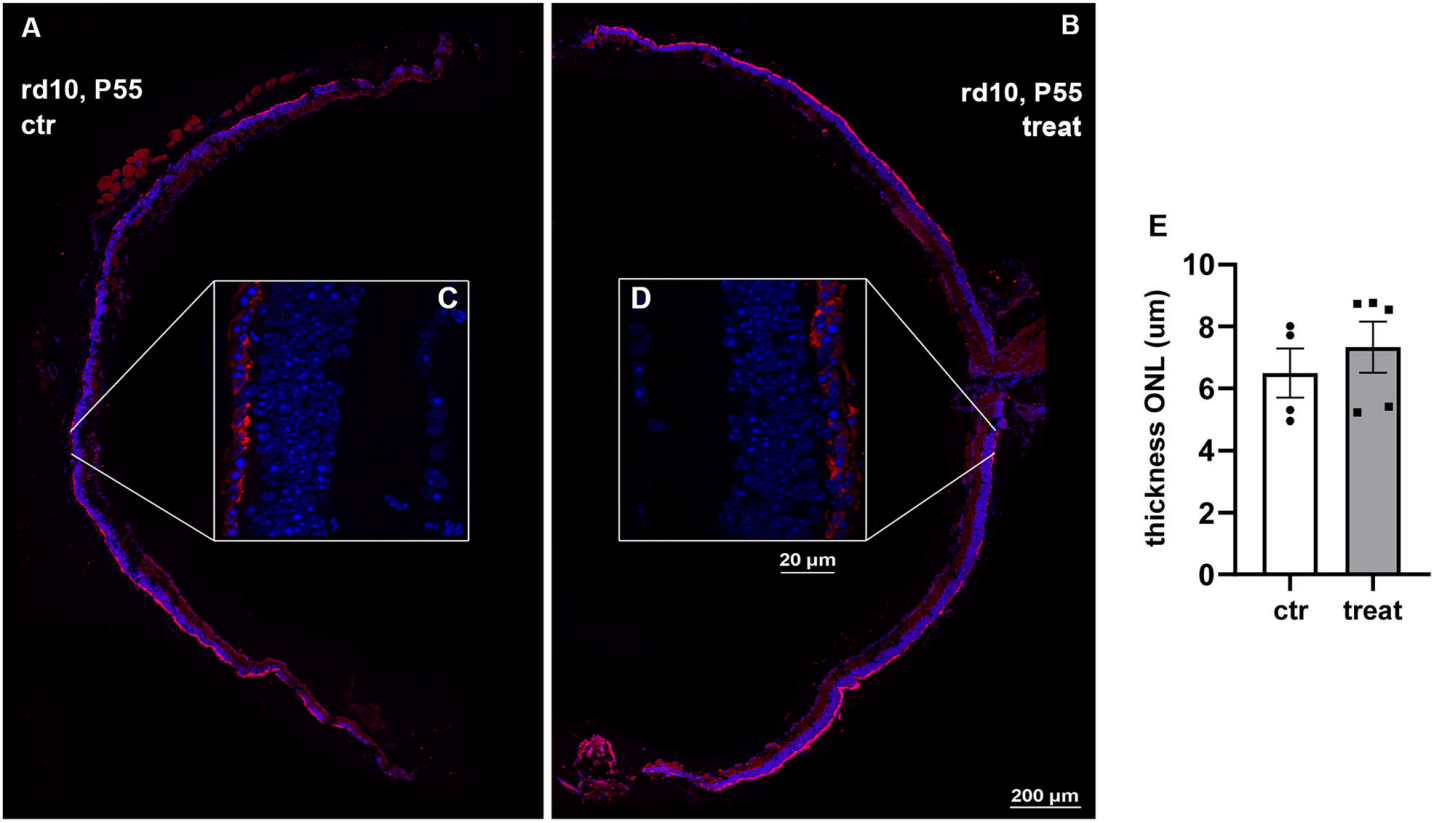
Retinal thickness is not rescued by intravitreal delivery of hNGFp in RP mice. ***A, B***, Representative images of the ctr (left) and treated (right) retinal section used to measure the thickness of the ONL. ***C, D***, zoom of retinal sections. ***E***, Statistical analysis of ONL measurements shows no changes in thickness upon hNGFp administration (ctr = 4, treat = 5: *p* = 0.495). Error bars are ±SEM.

To maximize retinal availability of hNGFp, the molecule was directly injected intravitreally, testing the effects on cone survival and retinal gene expression through immunocytochemistry and qPCR (Fig. 2*A*). It is generally not feasible to repeat multiple ocular injections because of the high risk of damaging the small mouse eye; hence, the animals were injected once at P50 and examined after 5 d. As a rule, retinas of treated and control animals were stained with cone arrestin antibodies; cone arrestin–positive photoreceptors were counted in retinal whole-mount using *z*-stacks of high-resolution images acquired along the width of the outer retina; the density and absolute cone number, as well as the area of maximum cone degeneration, clearly visible in fluorescence retinal whole mounts were measured and statistically compared. No differences between the control and the treated groups were visible (Fig. 2*B,D,E*). The thickness of the outer nuclear layer (ONL), where photoreceptor nuclei reside, was measured by using vertical retinal sections stained with cone arrestin antibodies and counterstained with a nuclear dye. The comparison between the treated and control groups showed a similar thickness of the ONL, with a trend in treated retinas to exhibit a slightly higher thickness, although not statistically significant (Fig. 3*A–E*). This suggests a moderate preservation of the photoreceptor layer due to hNGFp treatment. No differences in overall retinal areas were observed.

**Figure 4.**
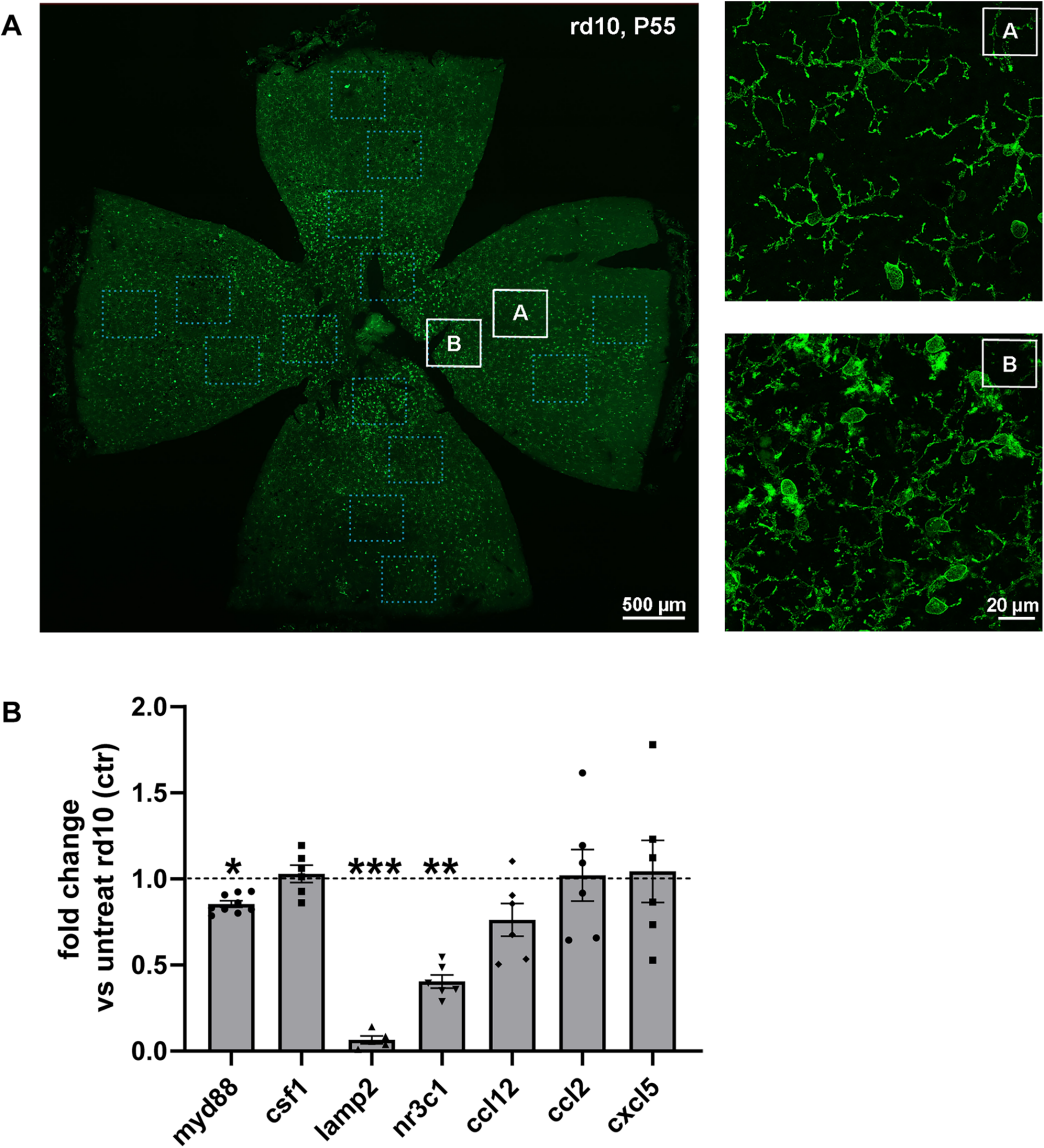
Intravitreal delivery of hNGFp shows partial immunomodulation of retinal inflammation in RP mice. ***A***, Whole-mount retinas of the control (left) and treated mice (right) stained with CD11b (green signal). The dotted lines define the degenerated area. ***A*** and ***B*** are zoom images of retinal microglia in the peripheral and central area **(*B***) qPCR quantification of genes involved in inflammatory responses**.** Myd88 (ctr = 8, treat = 9; *p* = 0.019) and csf1 (ctr = 5, treat = 6; *p* = 0.782) are transcriptional factors involved in the activation of pro- and anti-inflammatory response. Lamp2 (ctr = 5, treat = 6; *p* = 0.0005) is a protein involved in the autophagy process. Nr3c1 (ctr = 5, treat = 6; *p* = 0.002) codifies for glucocorticoid receptor expression. Ccl12 ctr = 5, treat = 6; *p* = 0.250), ccl2 (ctr = 5, treat = 6; *p* = 0.928) and cxcl5 (ctr = 5, treat = 6; *p* = 0.861) are chemokines and cytokines.

In accord with these observations, molecular analysis by single gene, real-time PCR revealed only a small but significant change in the expression of gnat2, a cone-specific, α subunit of transducin, and of ntrk1, the gene encoding for TrkA, involved in promoting neuronal survival (Fig. 2*C*). Among genes related to the inflammation pathway, expected to react to hNGFp administration, a highly significant increased expression was exhibited by tmem119, a type I transmembrane protein specifically expressed by homeostatic microglia at various levels in different pathological conditions (Ruan and Elyaman, 2022). The retinal expression of arg1 and nos2, two highly reactive markers of inflammation modulation, was also investigated (Stratoulias et al., 2019; Guo et al., 2022; Paolicelli et al., 2022). The results showed an expected trend, with increased arg1 expression and nos2 downregulation in hNGFp-treated retinas, although the observed differences between the control and treated groups were not statistically significant (Fig. 2*C*). This analysis extended to known key markers of the inflammatory innate responses, such as LAMP2 (lysosome-associated membrane protein-2), a glycoprotein involved in biogenesis and phagocytic activity upregulated in activated microglia (Blank et al., 2018; He et al., 2020), and MYD88 (myeloid differentiation primary response 88), an immune adaptor that activates inflammatory responses through NF-κB (Syeda et al., 2015; Garces et al., 2020). Both markers were indeed downregulated by the hNGFp treatment. We also analyzed the expression of NR3C1 (glucocorticoid receptor gene), whose activity is known to promote anti-inflammatory effects, but we found an unexpected and significant downregulation of this gene (Escoter-Torres et al., 2019; Escoter-Torres et al., 2020). No changes were found in the molecular expression of cyto-/chemokines (CSF1, CCL12, CCL2, CXCL5; Fig. 4*B*), which instead were demonstrated to be downregulated by wtNGF in microglia primed with Aβ oligomers (Rizzi et al., 2018). Histologically, no changes were found in the extension of the central retinal area of maximum photoreceptor degeneration upon hNGFp administration (Fig. 2*D*). Accordingly, this area was populated by highly reactive immune cells Cd11B+, with an ameboid shape (Fig. 4*A,B*), while the peripheral retinal areas displayed ramified, homeostatic microglia (Fig. 4*A*), as shown before (Guadagni et al., 2015; O’Koren et al., 2019; Rashid et al., 2019; Bennett and Viaene, 2021; Paolicelli et al., 2022). Microglial activation accompanies the well-known center-to-periphery gradient of photoreceptor loss typical of many rodent models of RP.

Taken together, these results suggest that intravitreal hNGFp treatment, although able to promote changes in gene expression of some retinal inflammatory markers, is not sufficient to support the rescue of cones, when started at P50 across the window of maximum cone death.

### Increased dosage of intravitreally delivered hNGFp does not ameliorate late cone degeneration and retinal inflammation in RP mice

**Figure 5.**
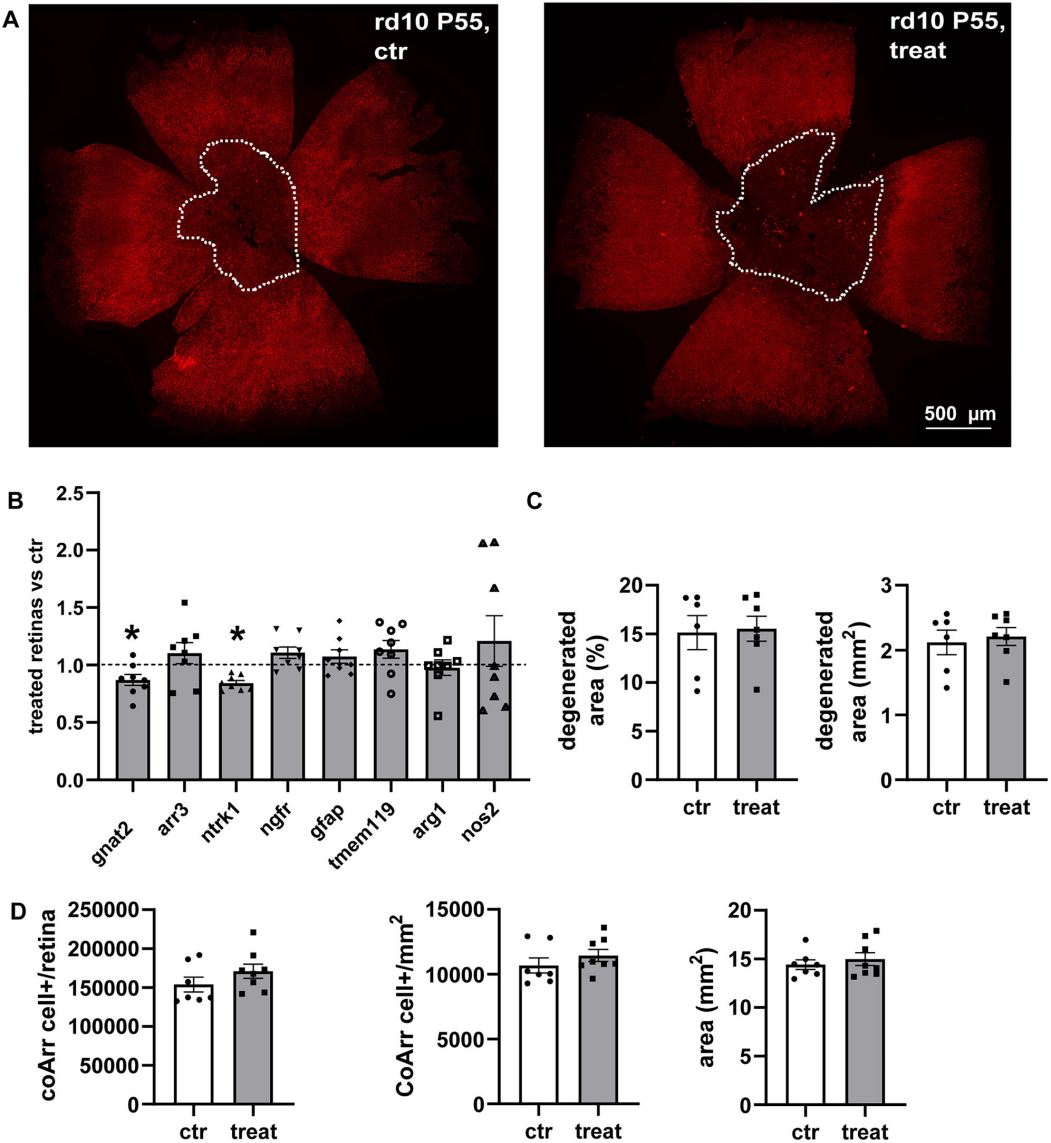
Increased dosage of hNGFp intravitreal delivered has no beneficial effects on cone survival and retinal inflammation of RP mice. ***A***, Whole-mount retinas of control (left) and treated mice (right) stained with cone arrestin (red signal). The dotted lines define the degenerated area. ***B***, qPCR quantification of genes involved in photoreceptors survival and microglial activation**.** Gnat2 (ctr = 7, treat = 8; *p* = 0.042) and Arr3 (ctr = 7, treat = 8; *p* = 0.486) are genes expressed in rods and cones, ntrk1 (ctr = 7, treat = 8; *p* = 0.050) is the gene for trkA expression while ngfr (ctr = 7, treat = 8; *p* = 0.092) is the one for p75NTR receptor expression. Gfap (ctr = 7, treat = 8; *p* = 0.500) refers to macroglial activation while tmem119 (ctr = 7, treat = 8; *p* = 0.270) represent the activation of microglia. Arg1 (ctr = 7, treat = 8; *p* = 0.831) and nos2 (ctr = 7, treat = 8; *p* = 0.415) are genes expressed by microglial cells. ***C***, Degenerated area quantification expressed as mm2 (ctr = 6, treat = 7; *p* = 0.698) and percentage (ctr = 6, treat = 7; *p* = 0.858) with respect to the total retinal area. ***D***, Quantification of cone arrestin–positive cells. Absolute cone number per retina (ctr = 7, treat = 8; *p* = 0.221); cone density (ctr = 7, treat = 8; *p* = 0.316); and retinal area (ctr = 7, treat = 8; *p* = 0.519). Error bars are ±SEM. *t* test was used to compare the ctr versus treat mean for each experiment.

To rule out the possibility that the hNGFp limited effect was due to a lower than necessary dose being administered, the dosage injected intravitreally was increased 100×, and the retinas of treated and control animals were analyzed as for the previous groups (Fig. 5). However, this dosage did not induce beneficial effects to the phenotype of the rd10 retinal degeneration mice. The expression of one of the two photoreceptor-specific tested genes, gnat2, was slightly but significantly decreased while arr3, the other tested gene, did not change. A downregulation of the prosurvival NGF receptor trkA (ntrk1) was detected, while the p75NTR gene (ngfr) did not change. Macroglia was not affected by this treatment as gfap expression did not change. Tmem119, which at the lower intravitreal hNGFp dose displayed a highly significant increased expression, was not modulated at this higher dose. The related arg1 and nos2 genes (microglial markers) did not change as well. However, arg1 and nos2 showed an opposite expression trend with respect to the experiments with lower hNGFp doses, suggesting some worsening of retinal inflammation and degeneration (Fig. 5*B*). However, no changes in the extension of the central retina area of maximum photoreceptor degeneration were detected, and counts of cone arrestin immunostained cones did not demonstrate a further decline in cone survival (Fig. 5*A,C,D*).

These data indicate that even if hNGFp is increased by two orders of magnitude, a therapeutic effect on the RP retinal degeneration is not achieved. The possible engagement of p75NTR signaling by the much higher dose of hNGFp might be considered.

### Intravitreal administration of wild-type NGF and hNGFp has no different effects on cone degeneration

**Figure 6.**
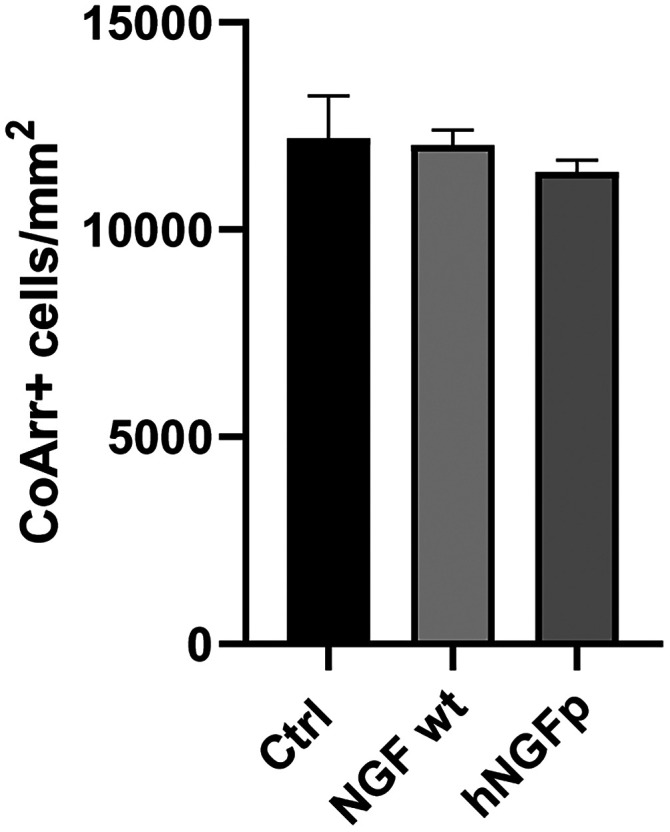
wtNGF and hNGFp treatments show no differences in cone survival. Cone counts in retinal preparations from rd10 mice injected intravitreally with wtNGF (*n* = 4), hNGFp (*n* = 6), and control solution (*n* = 6). Error bars are ±SEM. One-way ANOVA test. *p* = 0.7087.

To compare the effects of intraocularly injected hNGFp with those of wild-type NGF, currently used for laboratory studies and clinical treatments, wtNGF was injected intravitreally, at the same dosage used for hNGFp (0.54 ng/gr), in rd10 mice aged P50 and harvested retinal and RPE samples at P55; retinal and RPE morphologies were then compared with those obtained as a response to a back-to-back hNGFp (and control-vehicle) injection in mice of the same litter. No changes were detected in the overall survival rates of cones in wtNGF-injected samples, assessed by ICCH and cell count (Fig. 6), indicating that the molecular effectors of this drug, which are not identical to those of the painless hNGFp variant, also fail to elicit a cone-protective response in the rd10 mutant, in the experimental conditions used. Both upon wtNGF and hNGFp treatments, irregular areas of dense microglial activation persisted in the central retina, where microglial cells were observed to span both the remnants of the outer nuclear layer and the RPE. Highly activated cells, identified by short processes and ameboid shape, were recognizable in both experimental groups.

### Intranasal delivery does not improve hNGFp efficacy in RP mice

**Figure 7.**
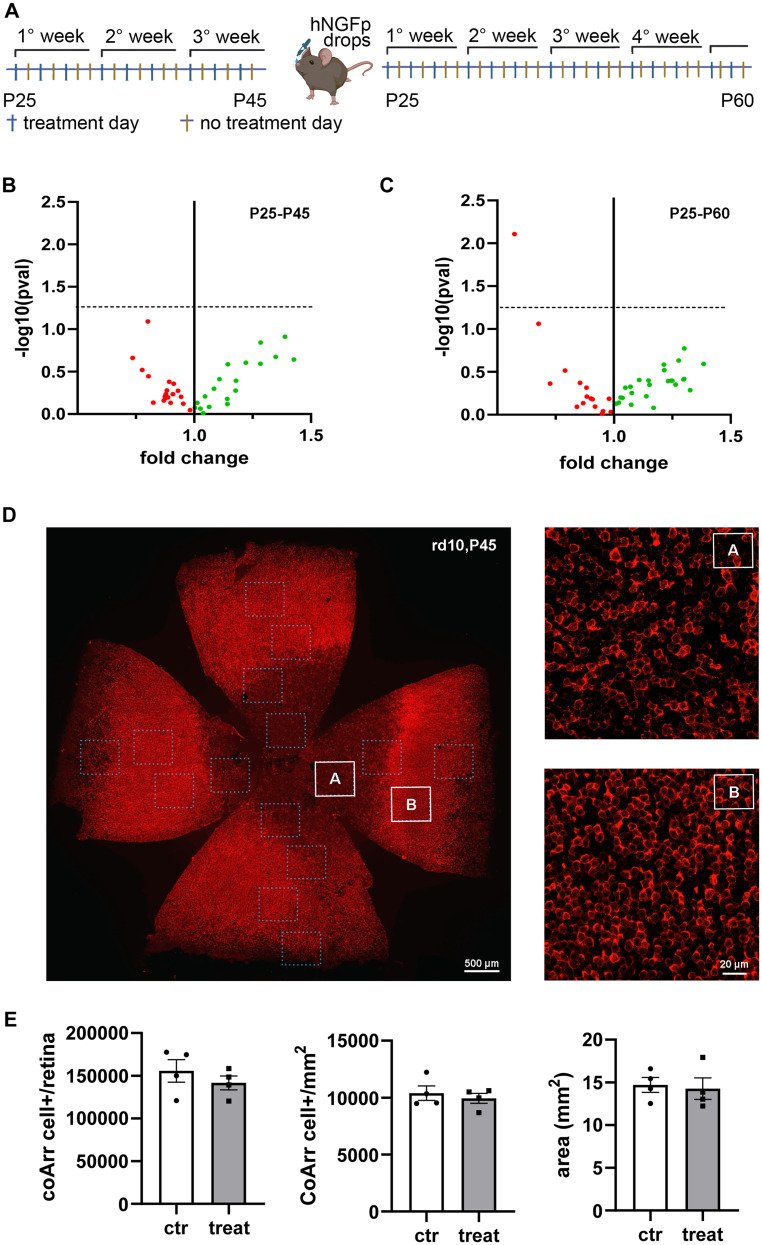
Intranasal delivery of hNGFp has no effect on cone rescue in RP mice. ***A***, Graphical representation of the experimental protocol adopted for the treatment. The animals were divided into two groups, control (ctr) and hNGFp treated (treat), and manipulated for 1 week before the start of the experimental protocol. Finally, they were intranasally treated with drops of PBS (ctr) or hNGFp (treat) 3 d/week from age P25 to P45 (left panel) or P60 (right panel). hNGFp dosage, 0.54 ng/gr. ***B, C***, Volcano plots of qPCR array show no changes of gene expression related to photoreceptor survival and the inflammatory pathway. The dots represent each tested gene with its mean fold change and the statistical value (-log10pval). The red and green dots are, respectively, the downregulated and upregulated genes in the treated animals with respect to the control mice. The dotted line represents the significance threshold [-log10(0.05) = 1.33.]. Volcano plot in ***B***, N:(ctr = 7, treat = 7), and in ***C***, N:(ctr = 6, treat = 6). ***D***, Whole-mount image (left side) of a rd10 retina (aged P45) stained with cone arrestin (red signal) showing how the acquisition for cone counting was done. The 16 dotted squares represent the investigated retinal areas for each animal while the white squares in ***A*** and ***B*** show zoom images of central and peripheral cone arrestin signals. ***E***, Quantification of cone arrestin signal. Absolute cone number per retina (ctr = 4, treat = 4; *p* = 0.399); cone density (ctr = 4, treat = 4; *p* = 0.582); and retinal area (ctr = 4, treat = 4; *p* = 0.781). Each dot represents a different animal. Error bars are ±SEM.

Having established that a single intravitreal delivery of either hNGF or hNGFp has no effect on the degeneration of cones, a possible rescue effect of hNGFp was tested using a sustained administration of the drug, beginning near the peak of rod degeneration. The intranasal way of delivery of hNGFp was chosen, for it was successfully employed in experiments on Alzheimer’s mouse models to target cholinergic neurons of the basal forebrain (BFCNs; Tiberi et al., 2024). This noninvasive route of administration allows repeated supplies of the drug and reaches, among others, both BFCNs in the brain and the external part of the eye through the nose lacrimal duct. Mice were treated with intranasal hNGFp three times a week, at the dosage of 0.54 ng/gr, or with vehicle solution for controls (Capsoni et al., 2012), starting at P25 up to either P45 or P60 (Fig. 7*A*). Afterward, the mice were killed, their eyes were collected, and the retinas were analyzed by qPCR arrays and immunohistochemistry as done for the intravitreal injection groups. A total of 43 different genes were analyzed through qPCR arrays, belonging to the (1) apoptotic pathway (bcl2, bax, bak); (2) inflammatory responses (gfap, cd68, nfkb, lamp2, myod88, itgam, aif1, mmp2, arsa, gaa); (3) cytokine and chemokine production (TNFa, INFg, ccl12, ccl2, cxcl5); (4) gene markers of microglial phenotype switching (cybb, dhcr7, arg1, nos2); (5) photoreceptor survival markers (cngb1, gnat1, gnat2, arr3); (6) inflammation; and (7) endothelial activation. The magnitude of the changed expression (fold change) was plotted versus the statistical significance (-log10pval) for each gene in a scatter plot (Fig. 7*B*). No significant changes in the expression of these genes were observed. Retinal samples were processed also as whole mounts with cone arrestin antibody staining. Cone profiles were imaged across the retinal surface and counted, and then cone density and absolute cone number in hNGFp-treated and control mice were compared (Fig. 7*D,E*). These analyses revealed no differences between the two groups, confirming the lack of measurable biological effects on retinal degeneration of hNGfp delivered through intranasal administration.

To extend the treatment window, searching for an increase in hNGFp efficacy, the administration was prolonged to P60, when qPCR analysis of retinal gene expression was again performed (Fig. 7*C*). However, this new experimental scheme failed to indicate an amelioration in the rate of retinal cone loss. No significant changes in the treated animals in comparison with the control group were detected in the pattern of expression of the tested genes, except for a significant downregulation of the interferon-γ receptor (infgr), in agreement with an immunomodulatory rule of hNGFp. However, all together these data suggest that hNGFp delivered by intranasal route has no efficacy in decreasing cone degeneration with the experimental paradigm chosen.

**Figure 8.**
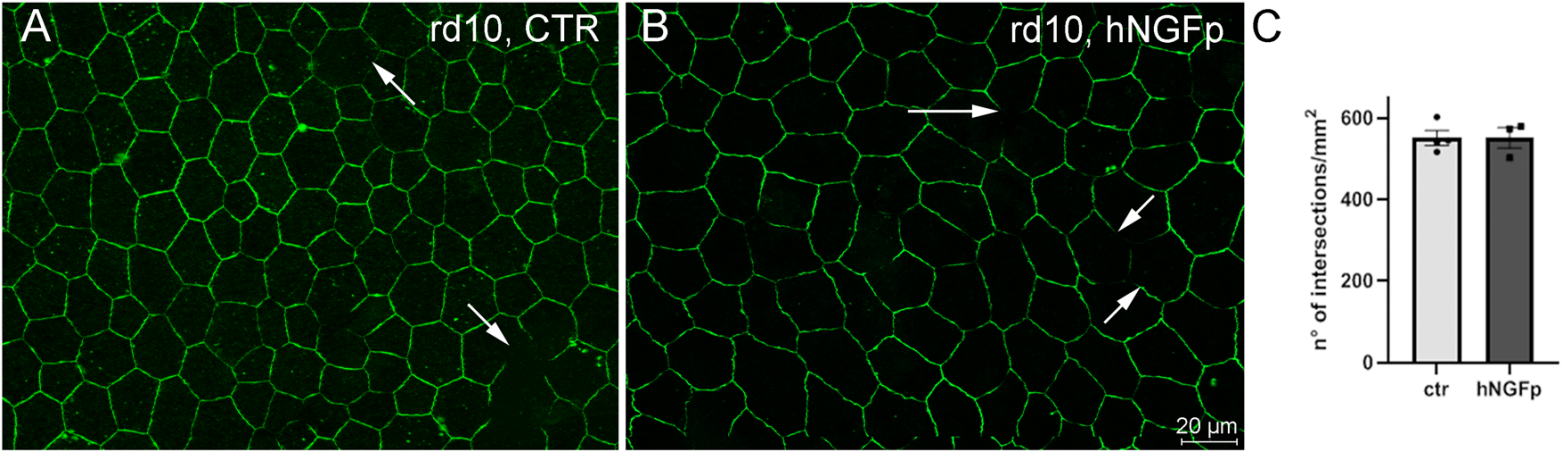
RPE analysis after intranasal delivery of hNGFp does not demonstrate structural amelioration. The administration failed to elicit visible effects on the morphology of the RPE, here shown upon immunolabeling with antibodies against ZO-1, a protein of the RPE tight junctional complexes. The number of discontinuities in the ZO-1 array (arrows), which is high in rd10 mutants as a secondary effect of photoreceptor loss (***A***), was not rescued by hNGFp administration (***B***), as demonstrated by a quantitative analysis shown in ***C***, where the number of intersections between ZO-1 profiles and a reference grid has been assessed systematically and compared statistically (*t* test; *p* = 0.62). Error bars are ±SEM. *t* test was used to compare the ctr versus treat mean for each experiment.

To disclose other potential biological targets of hNGFp concurring with the RP phenotype, we analyzed in the same animal of the P25–P45 treatment protocol the fine structure of the retinal pigment epithelium (RPE), among other functions, being the constituent of the outer blood–retina barrier (oBRB; Strauss, 2005; Yang et al., 2021). Zonula occludens 1 (ZO-1) is one of the most abundant proteins of the RPE tight junctions, and its distribution becomes discontinuous and strongly irregular when the oBRB is altered, as is the case of RP or other ocular diseases with a major inflammatory component (Rizzolo, 2007; Naylor et al., 2019; Napoli et al., 2021); these alterations can be prevented by treatments repairing the outer retina. We analyzed the RPE of the same rd10 mice used in the experiments described above by ZO-1 staining and quantitative analyses of RPE images, assessing the continuity of ZO-1–positive profiles (Fig. 8*A–C*). The comparison of RPE samples from hNGFp-treated and control mice showed no statistical differences in the pattern of ZO-1 staining, suggesting no effects of the drug on RPE cells.

### Intranasal delivery of hNGFp exerts a measurable effect on cholinergic neurons of the basal forebrain (BF)

**Figure 9.**
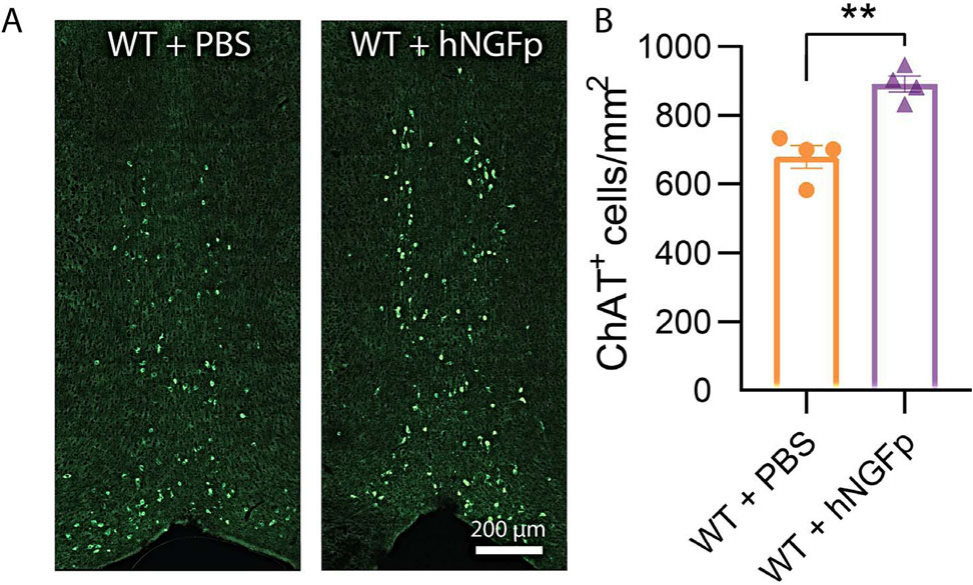
Biological action of the hNGFp used in this study. ***A***, Basal forebrain coronal sections. ChAT immunofluorescence staining. The number of ChAT-positive profiles is visibly more numerous in the hNGFp-treated section (right), compared with the control. ***B***, Cell counts on multiple sections show an approximate one-third increment in the number of ChAT-positive cells in hNGFp-treated samples (error bars are ±SEM. *t* test was used to compare the ctr vs treat mean for each experiment; **p ≤ 0.005).

Having established that the intraocular or the nasal delivery of hNGFp has no effect on the degeneration of cones in the rd10 mouse model, the in vivo biological efficacy of the batch of hNGFp used in our study was tested in parallel experiments in which the drug was administered by intranasal route to wild-type mice, according to the same protocol used in the rd10 model of RP. Immunocytochemical staining of BF cholinergic neurons with anti-choline acetyltransferase (ChAT) antibodies, followed by cell count with ImageJ and statistical analysis, showed a significant increment in the number of ChAT-positive profiles in hNGFp-treated versus control mice, administered vehicle only (Fig. 9*A,B*). This confirms previous studies showing that the used drug, delivered by the intranasal route, has an effect on BF neurons and provides a positive control of the biological activity of the stock of hNGFp employed for retinal studies.

## Discussion

Typical RP is characterized by a slow degeneration of photoreceptors in which rods die due to genetic mutations, while cones degenerate because of the detrimental environment that rod death creates. Given the extremely high genetic heterogeneity of RP, mutation-independent approaches to prevent or slow down the disease by attenuating bystander, detrimental effects leading cones to death could be beneficial for vision preservation in a potentially high number of RP individuals. In the present paper, the potential of a variant of NGF has been tested for the first time as a treatment for retinal degeneration in a well-established mouse model of RP. From a pharmacological point of view, hNGFp maintains the same neurotrophic and neuroprotective activity of the wtNGF, combined with a minimal nociceptive effect and a decrease of other p75NTR-related signaling effects (Cattaneo and Capsoni, 2019; Capsoni and Cattaneo, 2022). In addition, immunomodulatory properties of hNGFp have been recently discovered making it well suited to potentially treating RP (Capsoni et al., 2017; Lisi et al., 2022; Tiberi et al., 2024), where decrement of survival factors and inflammatory responses contribute to the progression of the disease. This study specifically analyses the effect of hNGFp on cone secondary degeneration and microglial activation in rd10 mutant mice by intranasal and intravitreal delivery. The nasal cavity, rich in blood vessel microvilli and branches of the trigeminal nerve, represents a noninvasive route to deliver drugs to the central nervous system, of which the retina is an extension; albeit still few studies have used this route to reach the retina, the peculiar anatomical conformation of the nasal cavity should ensure rapid absorption and fast onset of action (Hill et al., 2021; Ong et al., 2022; Patel et al., 2022). However, hNGFp-treated retinae from rd10 mice do not show any change in a panel of gene expression that can suggest any effect (beneficial or detrimental) of the treatment, either following a short or longer duration of administration (P25–P45 and P25–P60). None of the 43 different genes belonging to the apoptotic pathway, inflammatory responses, and cytokine and chemokine production, as well as marker genes of microglial or endothelial activation and of photoreceptors survival probed by qPCR, showed detectable changes. Immunohistochemical analysis of retinal samples confirmed that no increment in cone survival occurred following intranasal hNGFp. Structural indicators of RPE preservation and oRBR integrity (Napoli et al., 2021; Napoli and Strettoi, 2023) did not vary as well.

Ocular delivery of wtNGF is well documented, but there are no studies testing its specific effect on cone survival in RP. First papers regarding the beneficial effect of the neurotrophic factors in RP degeneration were formulated in the 1990s: C3H mice were treated by intraocular mouse NGF, and later RGS rats were injected with adenovirus encoding an *ngf/cntf* fusion gene (Lambiase and Aloe, 1996; Cayouette and Gravel, 1997); in both cases, the ONL thickness of treated animals was greater than in the controls, and this effect was attributed to neurotrophic protection of dying photoreceptors. Lenzi et al. (2005) confirmed the positive effect of NGF treatment through the analysis of retinal thickness and the increased expression of other neurotrophic factors and trkA receptors in retinal homogenates. Correspondingly, the retinas of animals treated with antibodies against NFG degenerate faster than control mice, suggesting a relevant role of NGF in mediating photoreceptor survival (Lenzi et al., 2005). Finally, a direct effect of NGF was demonstrated on primary cultures of photoreceptors isolated from young rats (Rocco et al., 2015). When a human form of NGF was produced and tested in RCS rats, an increase of retinal thickness was detected in treated animals (with both the murine or human NGF) but not in the sole ONL; increased photoreceptors survival included by the treatment was detected by cytofluorimetric analysis and TUNEL assay (Sacchetti et al., 2017). Despite these encouraging results, a pilot clinical trial on RP patients who received ocular NGF drops for 10 d failed to demonstrate a therapeutic action of NGF on retinal function (Falsini et al., 2016a,b). However, the degenerative stage of the patients enrolled in the study was generally advanced, and none of the previous studies regarding NGF treatment have specifically targeted bystander cone degeneration. In the present study, rd10 mice were treated at advanced degenerative stage (P50–P55). Our analyses suggest that hNGFp may have only a moderate effect on cone survival and microglial activation in the rd10 mutant, despite a slight upregulation of cone markers (gnat2) and of NGF receptor trkA by real-time PCR. We cannot exclude a therapeutic effect of hNGFp if administered at the beginning of photoreceptor degeneration, i.e., around the third postnatal week in the rd10 mutant. However, in the experimental design used here, the date of administration was deliberately chosen to favor a targeting of cones, in the attempt to achieve a direct rescue of these cells, at a stage of largely completed rod degeneration.

A possible explanation of the hNGFp failure in promoting cone survival in late degenerative stages could be the imbalanced expression of its receptors. Advanced retinal degeneration is accompanied by an increased expression of the competitive and proapoptotic receptors, p75/NTR, while trkA is downregulated (Sheedlo et al., 2002; Mysona et al., 2014; Malik et al., 2021). Indeed, blocking the p75/NTR activity can rescue photoreceptors in RP (Platón-Corchado et al., 2017). Although this study does not investigate the expression level of the two NGF receptors, our previous NGS data (Guadagni et al., 2019) demonstrate that the rd10 mice have expression levels of p75/NTR similar to aged-matched wt animals and a significant downregulation of trkA. Present data measuring the expression of trkA in rd10 mice treated with hNGFp in comparison with the untreated rd10 mice suggest at least a partial rescue of trkA expression toward physiological conditions. However, this receptor could be damaged in a pathological environment by local oxidative stress in its regulatory sites, as demonstrated for diabetic retinopathy retinal degeneration, preventing its phosphorylation and thus the biological function (Ali et al., 2008, 2011). Given all these considerations, it is not surprising that even hNGFp at increased dosage fails to show neuroprotective effects if used in late degenerating conditions, as suggested by RNA expression results. This might possibly be due to the engagement of p75NTR signaling at this 100× higher dose of hNGFp. Finally, it should be underlined that while the success of NGF in preventing cone death should be mainly linked to its neurotrophic and antiapoptotic activity, many studies have recently highlighted the relevance of nonapoptotic photoreceptor death, including ferroptosis, pyroptosis, and parthanatos (Campochiaro and Mir, 2018; Tang et al., 2019; Newton and Megaw, 2020).

Locally activated microglia are known to erroneously phagocyte still viable photoreceptors, while their continuous release of cytokines and chemokines becomes toxic for retinal cells contributing to chronic inflammation (Zhao et al., 2015; Guadagni et al., 2019; Ortega and Jastrzebska, 2021). In line with the homeostatically anti-inflammatory properties of hNGFp, a reduced expression of different inflammatory genes such as lamp2, myd88, and nr3c1 has been detected. The large increase in treated mice, of tmem119 expression, a microglial marker, might appear counterintuitive. However, recent in vitro studies show that two different types of human microglia exposed to hNGFp react differently with respect to iNOS and Arg1 expression and nitrite and urea production, respectively, considered pro- and anti-inflammatory indicators. These data suggest that different lines of microglia can react differently to hNGFp (Lisi et al., 2022), something that could also take place in the retina, where the occurrence of different types of microglia has been recently demonstrated (O’Koren et al., 2019). The differential expression of TMEM119 in different subpopulations of microglial cells has also been recently described and discussed (Ruan and Elyaman, 2022).

Particularly, the inner and outer retina microglia, dealing with the inner retina and photoreceptors homeostasis, respectively (McMenamin et al., 2019), might have different sensitivity to neurotrophic factors, so that each microglial plexus is better “tuned” to specific pleiotropic molecules that regulate neuronal development and survival. A yet unknown biological diversity and specificity might contribute to rendering outer retinal microglial cells relatively insensitive to an elsewhere powerful factor such as NGF, for which, evidently, they are not “tuned,” unlike ganglion cells, which respond well to neurotrophins of the NGF group (Mesentier-Louro et al., 2019). In addition, resident microglia are not the only immune cells responsible for the inflammatory responses occurring in RP. The integrity of the outer and inner retinal barriers is compromised and other immune cells might reach the retinal comportment (Ma et al., 2017; Yu et al., 2020; Zhao et al., 2022; Saadane et al., 2023). Gene therapy expression of sCX3CL1, a soluble factor that downregulates the phagocytic activity of immune cells, and the genetic induction of a “not-eat me signal” on photoreceptors are both able to protect cones from degeneration even after the complete ablation of resident microglia (Wang et al., 2019, 2021). Finally, He et al. (2022) found an increased number of immune cells expressing typical markers of neutrophils (CD45^+^Ly6g^+^CD11b/c^lo^), NK cells (CD45^+^CD49b^+^), and CD4^+^ T cells (CD45^+^CD3^+^CD4) in RP degenerating rats.

The scenario of contributors to bystander death of cones is highly complex and difficult to control with one single factor, even a powerful one on central neurons like NGF. However, it is not excluded that, in the future, this molecule or its hNGFp derivative, might reach a therapeutic effect if provided to the outer retina at an optimal dosage and with a chronic scheme (Wang et al., 2023) or with an earlier treatment.

## Synthesis

Reviewing Editor: Eran Perlson, Tel Aviv University Decisions are customarily a result of the Reviewing Editor and the peer reviewers coming together and discussing their recommendations until a consensus is reached. When revisions are invited, a fact-based synthesis statement explaining their decision and outlining what is needed to prepare a revision will be listed below. The following reviewer(s) agreed to reveal their identity: Ivan Anastassov, Kate O'Neill.

This study contributes to our understanding of effective therapies for RP. The authors investigate the potential protective effects of delivering a specific variation of the NGF neurotrophic factor, hNGFp, to a classical mouse model of retinal degeneration, rd10. They explore the effect of hNGFp introduction to the retina via two different methods and specifically address whether cones are spared from bystander degeneration after rod death. Despite the careful design and execution of the experiments, the results indicate that hNGFp does not have a protective action on surviving cones, as evidenced by immunofluorescence and qPCR analysis.

While the study has negative results, it still offers valuable insights to the field.

Several points are necessary for clarity and completeness before publications:

1. Provide higher-resolution images in Fig. 2B and 2C to facilitate better visualization of the degenerated and non-degenerated areas. Additionally, clarify the representation of gene expression levels in Fig. 2D.

2. Elaborate on the methodology for measuring ONL thickness in Fig. 3 to ensure consistency across conditions. Specify whether measurements were taken at multiple locations and whether data points represent averages.

3. Expand on the interpretation of results in Fig. 4, particularly regarding the downregulation of specific genes. Provide additional insights or cite relevant literature to support the findings.

4. Discuss the potential effects of hNGFp at earlier stages of degeneration, considering the concept of a "point of no return" for cones. Address whether hNGFp administration earlier in the disease progression could yield different outcomes.

5. Explore the consistent differences in morphology observed in peripheral and central microglia in Fig. 4. Provide further elaboration or cite relevant literature to contextualize these findings.

6. Correct the typo in line 496 for clarity.

7. Clarify whether the magnification in Fig. 8 is consistent across panels A and B. Address any discrepancies in RPE cell densities between conditions.
